# *In Vitro* Cytokine Expression and* In Vivo* Healing and Inflammatory Response to a Collagen-Coated Synthetic Bone Filler

**DOI:** 10.1155/2016/6427681

**Published:** 2016-04-18

**Authors:** Daniele Bollati, Marco Morra, Clara Cassinelli, Saturnino Marco Lupi, Ruggero Rodriguez y Baena

**Affiliations:** ^1^Nobil Bio Ricerche S.r.l., Via Valcastellana 26, 14037 Portacomaro, Italy; ^2^Department of Clinico-Surgical, Diagnostic and Pediatric Sciences, School of Dentistry, University of Pavia, P.le Golgi 2, 27100 Pavia, Italy

## Abstract

The goal of the present work was to investigate the relationship between* in vivo* healing and inflammatory response and* in vitro* cytokine expression by macrophages of a synthetic bone filler (25% hydroxylapatite-75% *β*-tricalcium phosphate) bearing a surface nanolayer of collagen. A clinically accepted, state-of-the-art xenograft material was used as a “negative control,” that is, as a material that provides the correct clinical response for the intended use.* In vitro* data show that both materials exert a very low stimulation of proinflammatory cytokines by macrophages, and this was confirmed by the very mild inflammatory response detected in* in vivo* tests of local response in a rabbit model. Also,* in vitro* findings suggest a different mechanism of healing for the test and the control material, with a higher regenerative activity for the synthetic, resorbable filler, as confirmed by* in vivo* observation and literature reports. Thus, the simple* in vitro* model adopted provides a reasonable forecast of* in vivo* results, suggesting that new product development can be guided by* in vitro* tuning of cell-materials interactions.

## 1. Introduction

In many cases dental implant therapy requires bone regeneration procedures through bone graft materials. In fact, the lack of dentoalveolar bone can disallow the therapy by compromising primary implant stability [1, 2]. To be clinically effective, the graft material does have not only to replace the missing tissue, but also to reinforce the injured area through the stimulation of two main processes [3]:

1. A “natural” healing mechanism, in which inflammatory cells behavior can be modulated to result in an advantageous biological local environment.
2. New bone ingrowth into the defect site, which should penetrate and replace the graft enabling the optimal balance between form and function.

While most of the published papers on bone regeneration through graft materials involve bone cells behavior and new bone formation, inflammatory response at the implant site and its correlation with bone formation and resorption should also be investigated. There is indeed an increasing literature about osteoimmunology, namely, the cross talk between cells from the immune and skeletal systems [4–9]. The molecular mechanisms through which inflammatory signals can be “translated” to become “understandable” for the bone system are related to the expression of RANKL, OPG (competitive ligands expressed by both inflammatory cells and mesenchymal and osteoblast/stromal cells), and RANK (transmembrane receptor for RANKL and OPG, expressed in osteoclast precursor cells and mature osteoclasts) [6]. The expression ratio RANKL/OPG is a fundamental value to shift the balance between osteoblast and osteoclast activity and to drive the overall pathways towards bone formation or its resorption. The interaction of RANKL with its receptor RANK serves as a chemotactic and survival factor for osteoclasts [7, 8] and its expression is upregulated by molecules like IL-1*β*, IL-6, TNF-*α*, and prostaglandin E2 and other cytokines produced during the inflammatory response [10–12].

Since there are many shared molecular signaling pathways, bone balance (and increased bone formation) is influenced also through the modulation of inflammatory response to implant materials and devices. Very few papers describe more in detail inflammatory cell behavior and response upon contact with bone fillers in terms of gene and protein expression: Lange et al. [13] quantified cytokine expression upon contact with hydroxyapatite or tricalcium phosphate particles in peripheral blood mononuclear cells (PBMCs). They found that HA induces a greater GM-CSF and RANKL expression than *β*-tricalcium phosphate, suggesting a possible role for HA particles in bone loss after surgical hip replacement. Zerbo et al. [14] analyzed the effect of porous *β*-tricalcium phosphate particles after sinus floor augmentation in human patients. In this case they immune-stained histological sections for both osteoblast (Cbfa, BSP, and OPN) and osteoclast (TRAP) markers, demonstrating that TCP particles attract osteoprogenitor cells that migrate and differentiate into mature bone cells.

The goal of the present work was to investigate the relationship between* in vitro* cytokine expression by macrophages and* in vivo* healing and inflammatory response of a recently described bio-enhanced synthetic bone filler. The latter is a biphasic phosphate ceramic [15] featuring a nanolayer of cross-linked collagen type I on the granule surface, and it will be coded SB in the rest of this paper.* In vitro* and* in vivo* results confirm that SB stimulates enhanced bone regeneration as compared to the uncoated ceramic, confirming that the surface collagen nanolayer [16] supports and cooperates with the scaffolding effect of ceramic particles, in agreement with reported findings on the role of interfacial interactions between type I collagen on implant devices and bone cells and bone regeneration. In particular, the aim of the present study was to evaluate* in vitro* expression of several cytokines by both macrophages (J774A.1) and osteoblast-like SaOS-2 cell grown on SB and to correlate the results to those obtained in an* in vivo* animal model (rabbit), in which healing tissue and inflammatory response were inspected. Both cell lines used are continuous, not fastidious, and comparatively not sophisticated. As such, it is well known that they can be poorly representative of the actual behavior of corresponding primary cells [17]. Conscious of these shortcomings, it is however our effort to try to develop simple* in vitro* models that can be predictive of* in vivo* behavior. The ultimate goal is to fully implement biodesign of new implant materials, where product development is guided by* in vitro* tuning of cell-materials interactions.

In the present study, SB behavior is gauged against the widely used xenograft (bovine derived hydroxyapatite) Bio-Oss (from Geistlich Biomaterials). The latter is clinically considered the golden standard for periodontal and dentoalveolar surgery during bone augmentation procedures and owes its properties to the scaffolding effect prompted by the microarchitecture of the pristine bovine bone tissue [18–20]. A number of studies describe the interactions of Bio-Oss (BS in the rest of this paper) with the surrounding environment, cell attachment, proliferation, and gene expression [21–26]. Within the scope of this paper it can be considered as a “negative control,” that is, as a standard material that is known to exert a correct response in terms of clinical outcome, to be compared with the test material SB.

## 2. Materials and Methods

### 2.1. Biomaterials

The following materials were tested:

1. Synthetic bone filler (SB) based on 25% hydroxylapatite-75% *β*-tricalcium phosphate granules, 0.3 to 1 mm size range, bearing a surface cross-linked nanolayer of collagen from porcine source (TheraCol, Sewon Cellontech Co., Ltd., Korea); further information is reported in [16].
2. Bio-Oss, xenograft from bovine bone, 0.25–1 mm granule size, which was obtained from Geistlich.

Both materials were sterile, supplied in sealed vials containing 0.5 g of granulate material.

### 2.2. Gene Expression Experiments

The expression of cytokines and other inflammatory markers was assessed using the real time reverse transcription polymerase chain reaction (qRT-PCR).

In particular, granulated samples were layered on the bottom of sterile 12-well polystyrene culture plates (12-well multiwell plates, Cell Star, Greiner One*™*). To form a complete layer, about 0.40 g of granules was required.

A suspension of 1.25 ± 0.12 × 10^5^ J774A.1 macrophage cells, cultured in DMEM containing L-glutamine (Gibco, Life Technology S.r.l.), and 20% Fetal Bovine Serum (FBS Gibco, Life Technology S.r.l.), penicillin, streptomycin, and amphotericin B (Anti-Anti, Gibco, Life Technologies S.r.l.) was introduced into the wells containing the samples. Total RNA was extracted after 4, 24 h, and 72 h using MagMax Total RNA Isolation Kit (Life Technologies S.r.l.) following the manufacturer's instructions. In particular, the culture medium was removed by gently pipetting and 0.200 mL of the lysing buffer was applied to the cell layer growing on the granules. The lysis buffer was then gently recovered by pipetting, RNA quality was assessed by checking the *A*_260_/*A*_280_ ratio (1.6–2.0), and cDNA synthesized as reported below.

As for SaOS-2 osteoblasts cells total RNA was extracted after 24 h, 72 h, and 7 days. A suspension of 5.60 ± 0.19 × 10^5^ SaOS-2 osteoblast-like cells from human osteosarcoma, cultured in McCoy's 5a (Gibco, Life Technologies S.r.l.), containing 20% Fetal Bovine Serum (FBS Gibco, Life Technologies S.r.l.), penicillin, streptomycin, and amphotericin B (Anti-Anti, Gibco, Life Technologies S.r.l.) was introduced into the wells containing the samples.

Then total RNA was used as a template for cDNA synthesis using random hexamers as primer and Multiscribe Reverse Transcriptase (High Capacity cDNA RT Kit from Life Technologies).

cDNA amplification and relative gene quantification were performed using commercially available TaqMan probe and primers from Life Technologies. Full information on the used primers is available in the producer web site. Real time PCR was performed in duplicate for all samples and targets on a Step-One Plus instrument (Life Technologies) using the software Step-One, version 2.2. PCRs were carried out in a total volume of 20 *μ*L and the amplification was performed as follows: after an initial denaturation at 95°C for 10 minutes, the PCR was run for 40 cycles at 95°C for 15 s and at 60°C for 1 minute.

To normalize the content of cDNA samples, the comparative threshold (Ct) cycle method, consisting of the normalization of the number of target gene copies versus the endogenous reference gene GAPDH, was used. The Ct is defined as the fractional cycle number at which the fluorescence generated by the cleavage of the probe passes a fixed threshold baseline when amplification of the PCR product is first detected. For comparative analysis of gene expression, data were obtained by using the ΔCt method.

The sequences of the primers used are the following.

*Murine*:

 IL-1*β*: GTGCAAGTGTCTGAAGCAGCTATGG,
 IL-6: AGAAAAGAGTTGTGCAATGGCAATT,
 IL-10: CTGAGGCGCTGTCATCGATTTCTCC,
 TNF-*α*: TCCCCAAAGGGATGAGAAGTTCCCA,
 MCP-1: GCTCAGCCAGATGCAGTTAACGCCC,
 COX-2: GGACTGGGCCATGGAGTGGACTTAA,
 MCSF: AAAGGATTCTATGCTGGGCACACAG,
 GAPDH: ATGACAATGAATACGGCTACAGCAA.

*Human*:

 ALP: TACAAGCACTCCCACTTCATCTGGA,
 OPN: TGAGGAAAAGCAGAATGCTGTGTCC,
 RANKL: TATTTCAGAGCGCAGATGGATCCTA,
 OPG: GTGGTGCAAGCTGGAACCCCAGAGC,
 COX-2: GCTGGGCCATGGGGTGGACTTAAAT,
 mPGEs: CGGAAGAAGGCCTTTGCCAACCCCG,
 GAPDH: GGAGTCAACGGATTTGGTCGTATTG.

### 2.3. *In Vivo* Experiments

*In vivo* experiments were conducted at NAMSA (Northwood, OH 43619, USA), in accordance with the provisions of the FDA Good Laboratory Practice (GLP) Regulations, 21 CFR 58. NAMSA is an AAALAC International accredited facility and is registered with the United States Department of Agriculture. Additionally, NAMSA maintains an approved Animal Welfare Assurance on file with the National Institutes of Health, Office for Laboratory Animal Welfare. Review and approval by the NAMSA Ohio Division Institutional Animal Care and Use Committee (IACUC) were obtained prior to conduct of the study.

### 2.4. Experimental Method

The rabbit model is widely used for evaluating articles intended for clinical implantation. The lateral condyle of the femur provides a cancellous bone site, which will mimic the bone sites of clinical use. Experiments involved 22 New Zealand White male rabbits, with a body weight range from 3.5 kg to 4.0 kg at implantation and age approximately 7.5 months at implantation, with a minimum acclimation period of 6 days, identified by ear tags. Ten rabbits were included in the 12-week group and 12 in the 26-week group.

### 2.5. Pretreatment Procedures

The rabbits were weighed. For general anesthesia, each rabbit was injected intramuscularly with a mixture of ketamine hydrochloride and xylazine (34 mg/kg + 5 mg/kg) dosed at 0.6 mL/kg. A fentanyl patch (analgesic; 25 *μ*g/hr) was applied to an ear. For operative and postoperative analgesia, each rabbit was injected subcutaneously with 0.05 mg/kg buprenorphine (analgesic). Each rabbit received an intramuscular injection of the antibiotic enrofloxacin at 10 mg/kg. A veterinary ophthalmic ointment was applied to both eyes of each rabbit to protect the corneas from excessive drying. After the anesthetic had taken effect, rabbits were clipped free of fur over the lateral and medial aspects of the rear legs from the wing of the ilium to the tarsus. The surgical sites were scrubbed with a germicidal soap and wiped with 70% alcohol. The surgical site was painted with povidone iodine and draped. The rabbits were placed on isoflurane inhalant anesthetic for continued general anesthesia.

### 2.6. Implantation Procedures

The surgical site was draped. Using sterile technique, the lateral aspect of the distal end of the femur over the lateral condyle was exposed through a routine surgical approach. Following exposure of the bone, an initial pilot hole was created, using a drill with an approximate 1.5 mm bit, in the lateral aspect of the femoral condyle. Using a power drill with an approximate 4 mm drill bit, the hole was enlarged to approximately 4 mm in diameter. The defect had an approximate depth of 10 mm. A SB sample was implanted in the right femoral condyle and a BS control sample was implanted in the left femoral condyle of each rabbit. The samples were placed in the bone defect to fill the void and remain flush with the cortical surface. The fascia and subcuticular layer were closed with 4-0 absorbable suture and the skin was closed with surgical staples. The day of implantation was designated as Day 0.

### 2.7. Postoperative Procedures

Each rabbit was moved to a recovery area and placed on a heat source. Each rabbit was monitored for recovery from the anesthetic. Once sternal recumbency was achieved, each rabbit was returned to its cage. Each rabbit received another injection of the analgesic buprenorphine (0.05 mg/kg) at approximately 6 hours after the first injection. On Days 1–3, another dose of enrofloxacin was administered at 10 mg/kg.

### 2.8. Laboratory Observations

Rabbits were observed daily for general health. Wound clips were removed once incisions had healed. Body weights were recorded for all animals prior to implantation, weekly for the first 4 weeks, every 4 weeks thereafter, and prior to termination.

### 2.9. Terminal Procedures

At 12 weeks after implantation, ten rabbits were arbitrarily selected for termination. The selected rabbits were weighed and each rabbit was euthanized with an intravenous injection of a sodium pentobarbital based euthanasia solution. The bone implant sites and adjacent muscle tissue were examined macroscopically and the observations were recorded. Any adverse observations at the implant sites were described. Each femur was dissected free and removed. Femurs were cut as appropriate to allow the fixative to penetrate the bone tissue for proper fixation. The femurs were placed in 10% neutral buffered formalin (NBF). At 26 weeks after implantation, the remaining twelve rabbits were similarly euthanized, examined, and processed.

### 2.10. Histological Procedures

After adequate fixation, the defect sites with implants in place were removed by making transverse cuts through the bone proximal and distal to each implant site, taking care not to disturb the sites themselves. Each bone section was labeled to indicate its original location. The implant sites were processed for and embedded in Technovit for Exakt procedures. One slide from each block was prepared as a transverse section of the bone through the length of the defect and stained with hematoxylin and eosin. The identity (animal number; left/right and implant site) of each bone section was maintained during processing. The slides were provided to a pathologist for histological evaluation.

### 2.11. Evaluation

Macroscopic observations of the implantation sites were described and compared between SB and BS sites. A pathologist conducted the microscopic evaluation of the bone implant sites. The bone implantation sites were evaluated for tissue response and cellular reactions (including inflammation). Cellular changes were graded according to severity (0–4) based on the scoring scheme in ISO 10993, Part 6, Annex E. Representative images of implant sites were taken to demonstrate the microscopic findings.

## 3. Results

### 3.1. *In Vitro* Cell Results

#### 3.1.1. Macrophages

First, we evaluated the expression of several cytokines and inflammatory factors in J774A.1 macrophages by using a specific developed model of gene expression previously described [27]. Briefly, this model allows relatively quantifying the presence of endotoxins and contaminants on the surface of a given material through the expression of specific proinflammatory genes in J774A.1 macrophage cells. In particular, we evaluated the endotoxin-like response on the two granular materials by analyzing the expression of interleukin 1*β* (IL-1*β*) and interleukin 6 (IL-6) after 4, 24, and 72 hours of cell culture. According to this model, IL-1*β* and IL-6 are the most expressed lipopolysaccharide-induced cytokines [27, 28] and their expression is directly related to the amount of endotoxins on the surface of materials.

**Figure 1:**
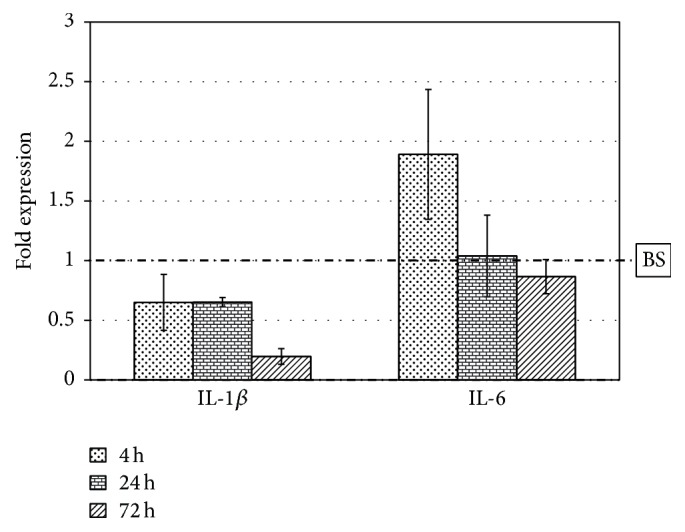
Interleukin 1-beta and interleukin 6 expression of J774A.1 macrophage cells grown on SB granules for 4 h, 24 h, and 72 h. Fold expression value is normalized to the expression on BS (dashed-dotted line).

Figure 1 shows the expression of the two genes on SB, compared to the expression on BS (dashed-dotted line). The expression of these interleukins does not significantly change (in terms of fold expression) between the two materials after 4 h and 24 h. These early time points are the most susceptible to the presence of endotoxins on the surface [27]: the detected expression was comparable to that of cells grown on tissue culture polystyrene (data not shown).

Interleukins are also implied in many signal pathways related to osteoclast differentiation: in particular IL-1*β* and IL-6 increase osteoclast formation through the induction of RANKL [10]. After 72 hours of cell culture we detected a significant decrease (about 5 times) of IL-1*β* expression on SB: this result could demonstrate that macrophage cells are less activated on SB than on BS granules. To fully elucidate these aspects the expression of several genes was analyzed to have a more complete understanding of inflammatory response. In particular four well known proinflammatory mediators were considered such as Tumor Necrosis Factor alfa (TNF-*α*), Macrophage Chemotactic Protein-1 (MCP-1), cyclooxygenase-2 (COX-2), and Macrophage Colony Stimulating Factor (MCSF) and an anti-inflammatory mediator like interleukin 10 (IL-10) [7, 10, 29, 30].

**Figure 2:**
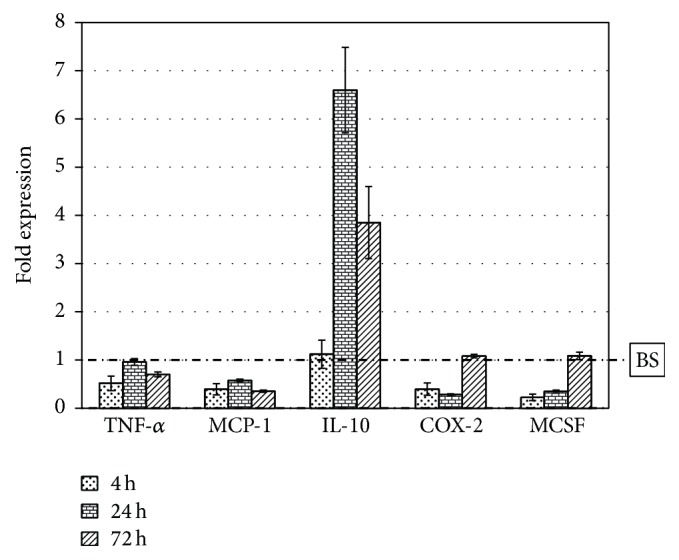
Gene expression of J774A.1 macrophage cells grown on SB granules for 4 h, 24 h, and 72 h. Fold expression value is normalized to the expression on BS (dashed-dotted line).

Figure 2 represents the expression of these genes on SB compared to BS: the surface-engineered synthetic biphasic material SB elicits a decrease in the expression of the four proinflammatory mediators at almost all the time points (up to 3-4-fold for MCP-1, COX-2, and MCSF), while maintaining unchanged (at 4 h) or increasing (at 24 h and 72 h) up to 5-fold the expression of the anti-inflammatory IL-10, compared to BS. These findings will be elaborated in the “Discussion.”

#### 3.1.2. Osteoblasts

Not only are cytokines and inflammatory signals produced by immune cells, but also osteoblast cells participate in their expression and/or stimulation. For this reason, the same samples were analyzed with SaOS-2 osteoblast cells and the expression of several genes was evaluated. In particular, the following genes were considered: ALP (alkaline phosphatase), a typical osteogenic marker [31, 32], OPN (osteopontin), an osteogenic marker also implied in inflammatory processes [33, 34], RANKL and OPG, fundamental proteins for bone remodeling and homeostasis [6, 7, 10, 11], and COX-2 and mPGEs (cyclooxygenase-2 and prostaglandin-synthase), two enzymes involved in the synthesis of prostaglandins, well known inflammatory mediators involved in bone metabolism and periodontal disease [35–37].

**Figure 3:**
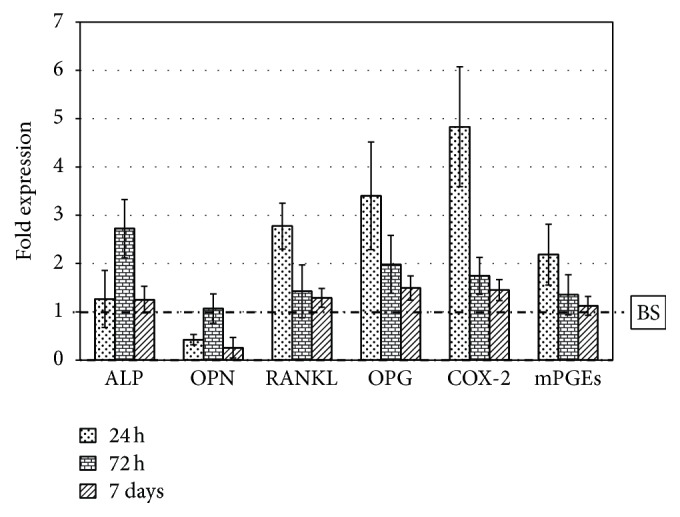
Gene expression of SaOS-2 osteoblast cells grown on SB granules for 24 h, 72 h, and 7 days. Fold expression value is normalized to the expression on BS (dashed-dotted line).

Figure 3 shows the obtained results: we observed a small general increase of the expression of these genes on SB compared to BS except for OPN, which is less expressed after 24 h and 7 days (about 3-4-fold). The genes involved in osteoblast-osteoclasts-immune cells communication tend to be more expressed especially after 24 h (about 3 times for RANKL and OPG and 5 times for COX-2), while at the other time points there are no significant differences (the values are close to 1, namely, the control BS). As for ALP expression, after 72 h we observed a significant increase for cells grown on SB granules (up to 3-fold).

### 3.2. *In Vivo* Results

**Figure 4:**
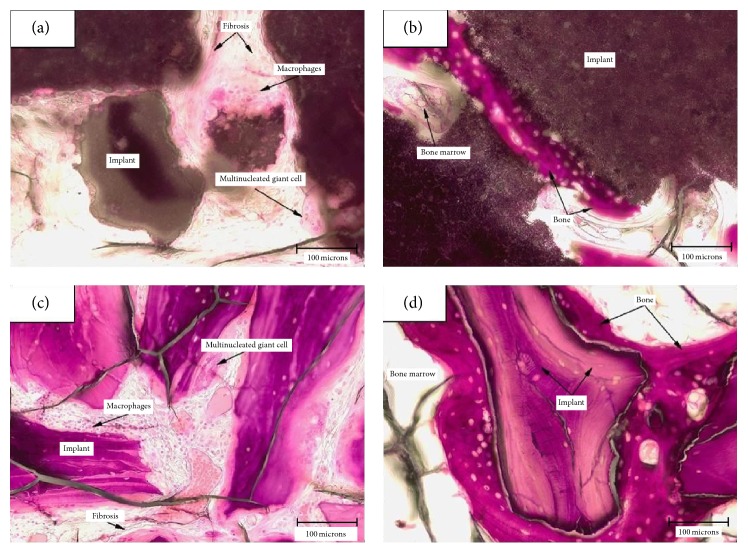
Microscopic evaluation after 12 weeks (20x objective). (a) Inflammatory cells and fibrosis surrounding the implant (SB) in the cortical area of the implant; (b) new bone has formed along the surface of the implant (SB) located in the medullary area of the defect without inflammation; (c) inflammatory cells and fibrosis surrounding the implant (BS) in the cortical area of the implant; (d) new bone has formed along the surface of the implant (BS) located in the medullary area of the defect without inflammation.

Figure 4 shows representative microscopic evaluation after 12 weeks: the low magnification image (Figures 4(a) and 4(c)) shows the defect largely filled, at this time point, by granules of the materials. A mild inflammatory response was observed and new bone formation was detected for both SB and BS (Figures 4(b) and 4(d)). The response was similar, although the irritancy score, according to the scoring scheme in ISO 10993, Part 6, Annex E, was slightly less for SB, as Table 1 shows.

**Figure 5:**
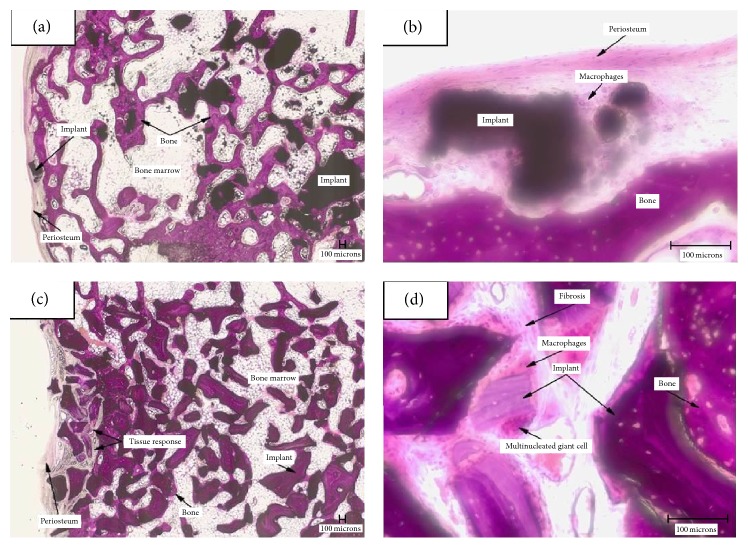
Microscopic evaluation after 26 weeks. (a) The majority of the granules (SB) are surrounded by trabecular bone in the bone marrow (2x objective); (b) granule of SB in the periosteum is surrounded by macrophages and there is a minimal thickening of the fibrous periosteum (20x objective); (c) the tissue response is prominent in the periosteum and cortical region of the implant (BS) (2x objective); (d) inflammatory cells and fibrosis surrounding the implant (BS) in the cortical area of the implant (20x objective).

After 26 weeks of implantation (Figure 5), trabeculae of new bone formed along many surfaces of the implanted SB and BS in all areas of the defect. In particular, granules of the biphasic SB have partially disappeared, and significant new bone formation is detected, with long trabeculae filling the defect area. BS granules are generally embedded in new bone, which bridges them. The inflammatory response was generally similar to that observed at 12 weeks with the exception that occasional focal accumulations of lymphocytes were observed around the implants in the bone marrow with both the products. Irritancy score was practically identical between the two products. Overall results show that bone is able to directly adhere to the surface of granules in both cases, and there are no local adverse effects and very few signals of any acute or chronic inflammatory response.

## 4. Discussion

Regeneration of bone defects results from an interplay of healing mechanisms, in which inflammatory cells behavior determines the biological local environment that supports new bone formation into the defect site. A recent work shows that the biomimetic approach adopted for the synthetic bone filler SB, by surface modification of ceramic particles through a type 1 collagen nanolayer, can promote more pronounced new bone formation as compared to conventional synthetic fillers, with a direct effect on osteogenic cells [16]. The present study aims at investigating inflammatory effect of the same material as related to bone regeneration. The expression of several key cytokines* in vitro* and* in vivo* response in a rabbit model was tested. The same tests were conducted on the golden standard BS, widely and satisfactorily used in clinical practice since years [21–23]. A limitation of this study is that just one cell line (macrophage) was used to test inflammatory cytokine expression, and it is of interest to evaluate whether* in vitro* data can nevertheless yield relevant information in the light of* in vivo* findings.

Considering cytokine expression by J774A.1 macrophages cultured on the tested materials, results, within the limits of this cell model, suggest first of all that both materials exert a very mild inflammatory response: cytokine expression is always comparable to that recorded on plain tissue culture polystyrene. An exaggerated cytokine expression could turn the peri-implant site into a proosteoclastogenic environment [38].

Importantly, data shown in Figure 1 show a very mild inflammatory response, comparable to that recorded on control polystyrene. Early time point data are most susceptible to the presence of endotoxins on the surface [27]; thus data in Figure 1 demonstrate that both materials are very “clean,” concerning endotoxins levels adsorbed on their surfaces. Hence, detected inflammatory response in these experiments is mostly dictated by genuine materials properties and not to spurious contributions from the “uninvited guest” [28].

Within this general result, data show that SB decreases the immediate inflammatory response of macrophages upon contact with granules and stimulates a different expression profile of cytokines compared to BS. One hypothesis could be that the monolayer of covalently linked collagen on the surface of the granules could act as a modulator of inflammatory response by mimicking the biological environment: this could lead to a decrease in the expression of proinflammatory signals and pathways related to the classical foreign body reaction. The overexpression of IL-10 on SB (Figure 2) is intriguing, because it has been demonstrated that IL-10 is more expressed in M2 activated macrophages (healer phenotype) than in classical M1 activated macrophages (inflammatory phenotype) [39]. An increase in its expression could then mean that a tissue healing mechanism is somehow encouraged by SB, again in agreement with known stimulation of healing response by collagen and surface-linked collagen layers [40–44].

The overall results on osteoblast cells demonstrate a rather similar response to the two materials. The increased expression of RANKL on SB is balanced by the same stimulation of the expression of osteoprotegerin: the RANKL/OPG ratio does not change; the higher expression recorded on SB could suggest increased bone remodeling activity. The enhancement of ALP expression at 72 hours is important because it could mean that cell differentiation is stimulated by SB, and they are induced to produce larger amount of mineralized bone, again in agreement with documented findings on collagen-coated bone-contacting devices [40–44]. The decrease of OPN expression is intriguing because of the connection of the role of osteopontin in inflammation processes: this role is still controversial but some studies [45, 46] demonstrate a direct control on macrophages migration and activation state in the early acute inflammation phases. Beside this function, osteopontin seems to facilitate the adhesion of osteoclast cells to bone matrix and to enhance their ability to migrate and resorb bone. Moreover, soluble osteopontin can sustain, through the mediation of its Arg-Gly-Asp (RGD) domain, the attachment of osteogenic cells to bone through their *α*_*v*_ *β*_3_ integrin receptor [46]. Finally a role for osteopontin in negatively regulating calcium phosphate crystal formation has been demonstrated both in soft tissues and during bone mineralization [47]. The decrease in its expression on osteoblasts grown on SB compared to BS could then represent a signal of a diminished stimulation of bone resorption.

*In vivo* results are in general agreement with* in vitro* findings: both tested materials show minimal inflammatory response; at 12 weeks the inflammatory score of SB, as evaluated by ISO10993 guidelines, is lower than that recorded on BS samples. So, the first conclusion is that the* in vitro* approach adopted, albeit involving continuous and nonsophisticated cell lines, has however some predictive power on* in vivo* response: BS is an accepted and satisfactory material; SB was not worse than BS* in vitro*; thus it is anticipated that SB would be equally satisfactory, at least from the point of view of the inflammatory response. This is confirmed by* in vivo* data.

At a more subtle level, the combined gene expression profile of macrophages and osteoblasts on SB suggests a different pathway for healing around SB as compared to BS implants: the decrease of the expression of the main proinflammatory cytokines, combined with the increase of the anti-inflammatory IL-10 in macrophage cells and the decrease of OPN expression together with the increase of ALP expression in osteoblasts, seems to stimulate a more appropriate inflammatory response to initiate a natural healing process and consequent new bone formation. This is understandable, because cells on SB do not face an inert material; rather they dialogue with the signaling cues of collagen type I.* In vivo* findings show indeed different evolutionary pathways of bone regeneration around the tested granules: in the case of BS, granules are not or very slightly resorbed, the material nicely fills the defect, and new bone embeds and bridges the granules (Figures 4(d) and 5(c)). The competitive and clinical advantage of BS over other materials could be that no high rate of new bone formation is required. The rate of new bone formation is appropriate to produce a regenerated bony bed made by newly formed bone that engulfs and bridges implanted granules. On the contrary, to be effective, SB requires enhanced rate of bone formation, because its *β*-tricalcium phosphate component is resorbed and the defect must be filled almost entirely by new bone (Figure 4(a)). This different behavior was already discussed in the literature in a different rabbit model of biphasic calcium phosphate versus BS bone regeneration experiment [48]. The different profile of cytokine expression detected* in vitro* underlines the onset of different and specific healing mechanisms, fit for the specific nature of both materials, hence the satisfactory results reported in the literature [16, 18–26]. A recent paper by de Lange and coworkers [49] compared gain of mineralized bone compared between deproteinized bovine bone allograft and biphasic calcium phosphate for dental implant placement following sinus elevation. The two different materials showed similar osteoconductive patterns and mineralized bone, although signs of more active bone formation and remodeling were observed in biphasic calcium phosphate—than in bone allograft—grafted biopsies. These findings are in agreement with the present suggestions.

## 5. Conclusions

The analysis of gene expression of several cytokines from simple* in vitro* models of SB and of the “negative control” BS gives indications that fit reasonably well with the picture arising from the evaluation of inflammatory response according to ISO 10993 standard and bone regeneration in a rabbit model. In particular, both materials promote a very mild inflammatory response. The bone allograft BS is very slightly resorbed and a regenerated bony bed made by newly formed bone that engulfs and bridges implanted granules is observed, while the collagen-coated biphasic calcium phosphate SB resorbs over time, and more active bone formation and remodeling is observed as compared to BS.

*In vitro* findings show, in general, mild expression of main proinflammatory cytokines by macrophages on both materials. The decrease of IL 1–6 expression, combined with the increase of the anti-inflammatory IL-10 by macrophages on SB as compared to BS, and decrease of OPN expression together with the increase of ALP expression in osteoblasts cultured on SB as compared to BS suggest indeed a more appropriate inflammatory response to initiate a natural healing process and consequent new bone formation on SB, according to* in vivo* findings and quoted literature reports.

The understanding, as attempted in the present work, of the relevance of different profile of cytokine expression* in vitro* in the light of specific mechanism of bone regeneration could be the basis of biodesign of novel and satisfactory implant materials.

## Figures and Tables

**Table 1:** 

	SB									
Rabbit number	78887	77888	78890	78891	78992	78886	78889	78897	78893	78907
Inflammation Polymorphonuclear	0	0	0	0	0	0	0	0	0	0
Lymphocytes	0	0	0	0	0	0	0	0	0	0
Plasma cells	0	0	0	0	0	0	0	0	0	0
Macrophages	0	0	0	0	1	1	0	1	1	1
Giant cells	0	0	0	0	1	1	0	1	1	1
Necrosis	0	0	0	0	0	0	0	0	0	0
Subtotal (*X*2)	0	0	0	0	4	4	0	4	4	4
Neovascularization	0	0	0	0	0	0	0	0	0	0
Fibrosis	0	0	0	0	1	1	0	1	1	1
Fatty infiltrate	0	0	0	0	0	0	0	0	0	0
Subtotal	0	0	0	0	1	1	0	1	1	1
Total	0^†^	0^‡^	0^‡^	0^‡^	5	5	0^‡^	5	5	5
Group total	2.5									
Traumatic necrosis	0	0	0	0	0	0	0	0	0	0
Foreign debris	0	0	0	0	0	0	0	0	0	0
Number of sites examined	0^†^	1	1	1	1	1	1	1	1	1

	BS									

Rabbit number	78887	77888	78890	78891	78992	78886	78889	78897	78893	78907
Inflammation Polymorphonuclear	0	0	0	0	0	0	0	0	0	0
Lymphocytes	0	0	0	0	0	0	0	0	0	0
Plasma cells	0	0	0	0	0	0	0	0	0	0
Macrophages	1	1	1	1	0	1	1	1	1	1
Giant cells	1	1	1	1	0	1	1	1	1	1
Necrosis	0	0	0	0	0	0	0	0	0	0
Subtotal (*X*2)	4	4	4	4	0	4	4	4	4	4
Neovascularization	0	0	0	0	0	0	0	0	0	0
Fibrosis	1	1	1	1	0	1	1	1	1	1
Fatty infiltrate	0	0	0	0	0	0	0	0	0	0
Subtotal	1	1	1	1	0	1	1	1	1	1
Total	5	5	5	5	0^‡^	5	5	5	5	5
Group total	4.5									
Traumatic necrosis	0	0	0	0	0	0	0	0	0	0
Foreign debris	0	0	0	0	0	0	0	0	0	0
Number of sites examined	1	1	1	1	1	1	1	1	1	1

^†^Implant was not present in the plane of section.

^‡^Implant was not present in the periosteum in the plane of section.
